# Hijacking of Host Plasminogen by *Mesomycoplasma* (*Mycoplasma*) *hyopneumoniae* via GAPDH: an Important Virulence Mechanism To Promote Adhesion and Extracellular Matrix Degradation

**DOI:** 10.1128/spectrum.00218-23

**Published:** 2023-05-18

**Authors:** Jiying Wang, Shiyang Li, Junhong Chen, Lanxi Gan, Jia Wang, Qiyan Xiong, Zhixin Feng, Quan Li, Zhibang Deng, Xiaomin Yuan, Yanfei Yu

**Affiliations:** a College of Veterinary Medicine, Hunan Agricultural University, Changsha, China; b Institute of Veterinary Medicine, Jiangsu Academy of Agricultural Sciences, Key Laboratory of Veterinary Biological Engineering and Technology, Ministry of Agriculture and Rural Affairs, Nanjing, China; c Guotai (Taizhou) Center of Technology Innovation for Veterinary Biologicals, Taizhou, China; d Department of Animal Science and Technology, Huaihua Polytechnic College, Huaihua, China; e School of Animal Science and Food Engineering, Jinling Institute of Technology, Nanjing, China; f Discipline of Microbiology, School of Life Sciences, University of Kwazulu-Natal, Durban, South Africa; g College of Veterinary Medicine, Yangzhou University, Yangzhou, China; University of Warwick

**Keywords:** *Mesomycoplasma hyopneumoniae*, GAPDH, adhesion, plasminogen, extracellular matrix

## Abstract

Mesomycoplasma hyopneumoniae is the etiological agent of mycoplasmal pneumonia of swine (MPS), which causes substantial economic losses to the world’s swine industry. Moonlighting proteins are increasingly being shown to play a role in the pathogenic process of M. hyopneumoniae. Glyceraldehyde-3-phosphate dehydrogenase (GAPDH), a key enzyme in glycolysis, displayed a higher abundance in a highly virulent strain of M. hyopneumoniae than in an attenuated strain, suggesting that it may have a role in virulence. The mechanism by which GAPDH exerts its function was explored. Flow cytometry and colony blot analysis showed that GAPDH was partly displayed on the surface of M. hyopneumoniae. Recombinant GAPDH (rGAPDH) was able to bind PK15 cells, while the adherence of a mycoplasma strain to PK15 was significantly blocked by anti-rGAPDH antibody pretreatment. In addition, rGAPDH could interact with plasminogen. The rGAPDH-bound plasminogen was demonstrated to be activated to plasmin, as proven by using a chromogenic substrate, and to further degrade the extracellular matrix (ECM). The critical site for GAPDH binding to plasminogen was K336, as demonstrated by amino acid mutation. The affinity of plasminogen for the rGAPDH C-terminal mutant (K336A) was significantly decreased according to surface plasmon resonance analysis. Collectively, our data suggested that GAPDH might be an important virulence factor that facilitates the dissemination of M. hyopneumoniae by hijacking host plasminogen to degrade the tissue ECM barrier.

**IMPORTANCE** Mesomycoplasma hyopneumoniae is a specific pathogen of pigs that is the etiological agent of mycoplasmal pneumonia of swine (MPS), which is responsible for substantial economic losses to the swine industry worldwide. The pathogenicity mechanism and possible particular virulence determinants of M. hyopneumoniae are not yet completely elucidated. Our data suggest that GAPDH might be an important virulence factor in M. hyopneumoniae that facilitates the dissemination of M. hyopneumoniae by hijacking host plasminogen to degrade the extracellular matrix (ECM) barrier. These findings will provide theoretical support and new ideas for the research and development of live-attenuated or subunit vaccines against M. hyopneumoniae.

## INTRODUCTION

Mycoplasmas are host-specific pathogens that cause chronic infections that are often difficult to eradicate. *Mesomycoplasma* (*Mycoplasma*) *hyopneumoniae* is the most important pathogenic mycoplasma in swine, being the etiological agent of mycoplasmal pneumonia of swine (MPS), with an average prevalence of 30 to 80% worldwide (1, 2). The pathology of M. hyopneumoniae is determined mainly by the damage caused by interactions between M. hyopneumoniae and host cells. M. hyopneumoniae infection leads to epithelial damage of the swine respiratory tract, which includes the loss of cilia, cell death, and a host inflammatory response. M. hyopneumoniae is a membrane-associated pathogen, and adhesion to the cilia of the respiratory epithelium is the initial event in host colonization. Hence, adhesion factors are usually considered to be the most important virulence factors. Members of the P97 and P102 adhesin families have been extensively studied and are considered typical adhesins: they can act as receptors on the cilia of the respiratory epithelium surface that bind to cilia-exposed glycans, swine extracellular matrix (ECM) molecules (such as heparin and fibronectin), and extracellular actin (3–6). It is also important that M. hyopneumoniae can bind and activate plasminogen (7). During infection with M. hyopneumoniae, plasmin levels in lung sections were elevated (5). Additionally, other less typical adhesins, such as some enzymes that moonlight as adhesion proteins, were reported in recent years. For example, NAD oxidase (NOX), NAD-dependent flavin oxidoreductase (NFOR), elongation factor thermo unstable (EF-Tu), and fructose-1,6-bisphosphate aldolase (FBA) are some adhesion molecules of M. hyopneumoniae that have been identified to date (8–12), and many more likely remain to be revealed.

The ECM is one of the most important physical barriers that restrict the spread of mycoplasmas, as reported for several mycoplasma species, including Mesomycoplasma hyorhinis, Mycoplasmoides pneumoniae, and M. hyopneumoniae (13–15). A common feature of invasive processes is that invasive cells migrate into adjacent tissues or the circulation via ECM degradation (16). Plasminogen is an important component of the fibrinolytic system, which is a proteolytic system that plays an important role in ECM degradation (17). Upon infection, mycoplasmal surface-bound plasminogen is transformed into plasmin, and the plasmin level in the airways of pigs has been shown to be increased, which turns the mycoplasma into a proteolytic organism via the fibrinolytic system (13, 18). The fibrinolytic system might facilitate its trafficking via the circulatory system, allowing it to penetrate other host organs. In fact, M. hyopneumoniae has been successfully isolated from the liver, kidney, and spleen (19, 20).

Previous studies have reported that glyceraldehyde-3-phosphate dehydrogenase (GAPDH) is a plasminogen receptor (PlgR) in M. hyorhinis that hijacks plasminogen on the mycoplasmal surface. The bound plasminogen can be transformed into plasmin by urokinase plasminogen activator (uPA) or tissue plasminogen activator (tPA), which initiates a proteolytic cascade that results in damage to tissue barriers (16). Thus, we speculated whether GAPDH is involved in the virulence of M. hyopneumoniae by hijacking plasminogen to transform it into plasmin, followed by the degradation of the ECM.

In this study, we compared the expression levels of GAPDH in highly virulent M. hyopneumoniae strain 168 and its attenuated highly passaged strain 168L, explored the atypical functions of GAPDH in the cytoadhesion of M. hyopneumoniae, and examined the interaction between the plasminogen/plasmin system and ECM degradation.

## RESULTS

### Differential abundances of GAPDH between pathogenic and attenuated M. hyopneumoniae strains.

**FIG 1:**
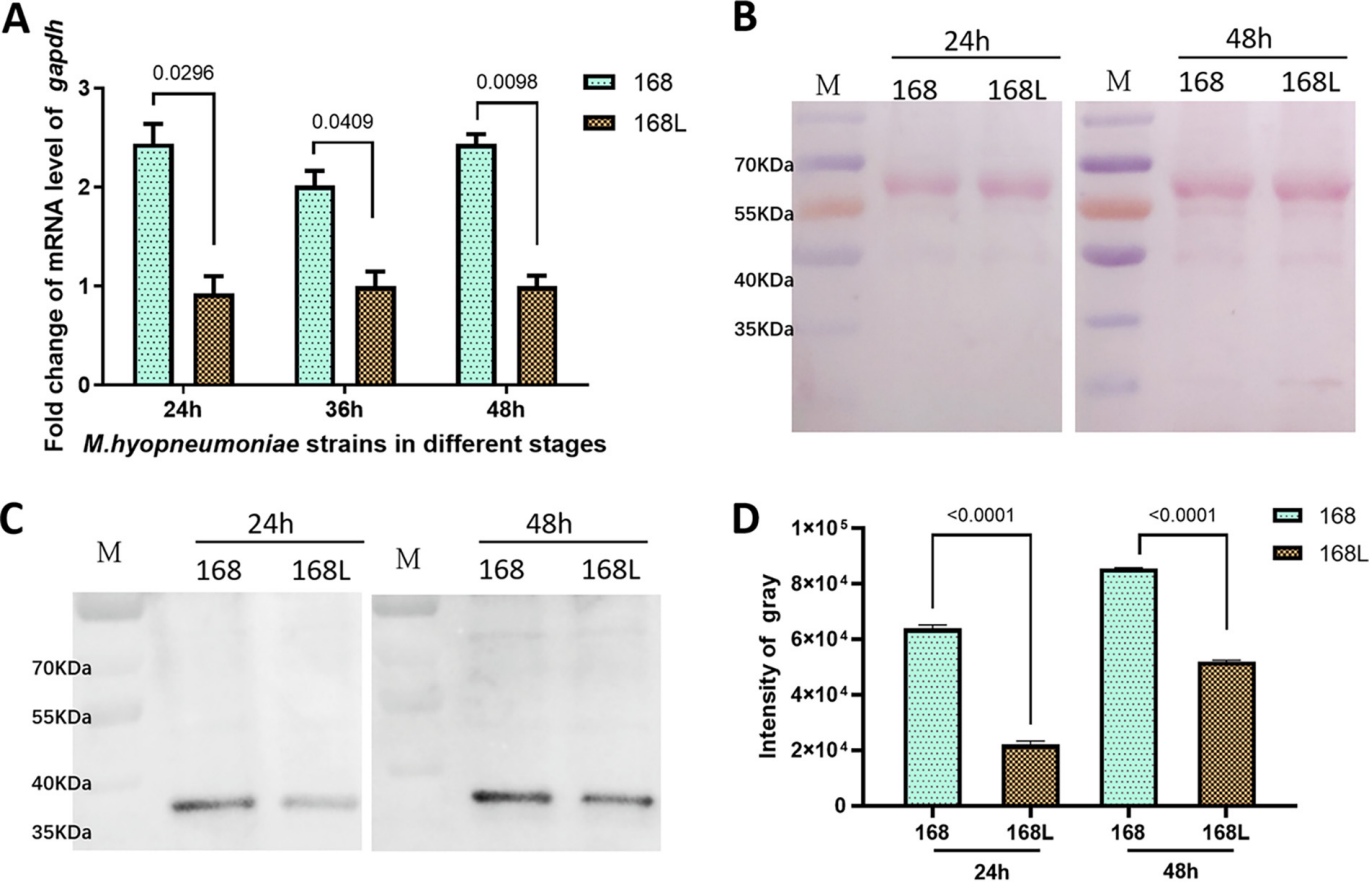
Expression levels of the GAPDH gene from virulent M. hyopneumoniae strain 168 and attenuated strain 168L. (A) Relative expression levels of the GAPDH genes from M. hyopneumoniae168 and 168L. Gene expression was determined using RT-qPCR analysis. Error bars represent standard deviations (SD) from three independent experiments. (B) Ponceau S-stained PVDF membrane. M, protein marker (in kilodaltons). (C) Differential abundance of GAPDH (37 kDa) analyzed using anti-rGAPDH sera. Protein bands were visualized using the ECL substrate. M, protein marker (in kilodaltons). (D) Intensity of the bands in Fig. 2C analyzed using ImageJ software.

Western blotting and reverse transcription-quantitative PCR (RT-qPCR) were used to evaluate the association of GAPDH with the virulence of M. hyopneumoniae. The results of RT-PCR showed that the level of GAPDH mRNA differed significantly between virulent M. hyopneumoniae strain 168 and its attenuated strain 168L. As shown in Fig. 1A, the mRNA abundance of the GAPDH gene was significantly higher in strain 168 than in strain 168L in the early (24 h), middle (36 h), and late (48 h) growth stages (*P* < 0.001). The results of Western blotting showed that the protein abundance was significantly higher in strain 168 than in strain 168L in the early and late growth phases (Fig. 1B to D). Thus, the two approaches showed a consistent upregulation of GAPDH in the virulent strain, preliminarily suggesting an association between GAPDH and M. hyopneumoniae virulence.

### GAPDH is partially located on the surface of M. hyopneumoniae.

**FIG 2:**
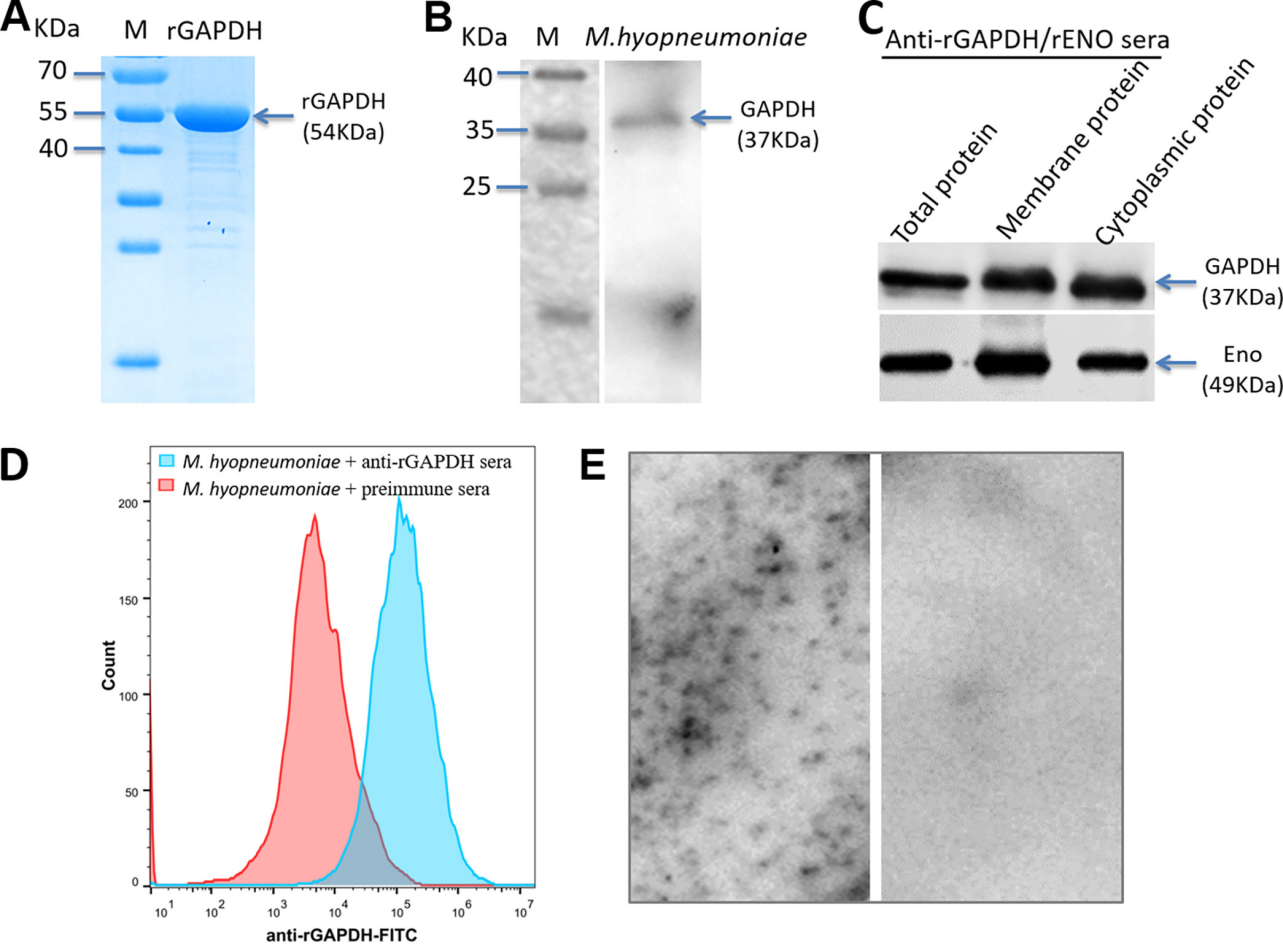
Cloning, expression, purification, and localization of GAPDH. (A) Analysis of rGAPDH (54 kDa) by 12% SDS-PAGE. M, protein marker. (B) Western blotting of M. hyopneumoniae proteins using anti-rGAPDH sera (diluted 1:1,000). M, protein marker (in kilodaltons). GAPDH is 37 kDa. (C) Subcellular localization of GAPDH in M. hyopneumoniae. First lane, whole-cell extract; second lane, membrane proteins; third lane, cytosolic proteins incubated with anti-rGAPDH sera or anti-recombinant Eno (rENO) sera (diluted 1:1,000). (D) Detection of GAPDH by flow cytometry. M. hyopneumoniae strains were incubated with anti-rGAPDH sera or negative sera. (E) Detection of surface GAPDH by colony blotting. The reaction of M. hyopneumoniae colonies transferred to a PVDF membrane and probed with anti-rGAPDH sera (left) or negative sera (right) was determined.

The GAPDH gene is very conserved among different strains of M. hyopneumoniae. The full-length GAPDH gene, designed based on the genome sequence of strain 168, was synthesized and cloned into the prokaryotic expression vector pET-32a. Recombinant GAPDH (rGAPDH) was expressed in Escherichia coli BL21(DE3) and purified. Purified rGAPDH was detected by sodium dodecyl sulfate-polyacrylamide gel electrophoresis (SDS-PAGE) as a band of ~54 kDa, which corresponded to the size of GAPDH with a His tag (Fig. 2A). Anti-rGAPDH sera specifically reacted with GAPDH of M. hyopneumoniae, represented by a single clear band at 37 kDa in the whole bacterial protein sample (Fig. 2B). Enolase (Eno) is a well-known surface protein of M. hyopneumoniae (21), which was used as a positive control to examine the cellular localization of GAPDH. As shown in Fig. 2C, both GAPDH and Eno were detected in the whole-cell extract, membrane protein, and cytosolic protein fractions of M. hyopneumoniae (Fig. 2C). Mycoplasmas do not have a cell wall such that their cell membrane components will interact with the host as the first step during infection. Flow cytometry and colony blotting were then used to further verify whether GAPDH is present on the surface of the M. hyopneumoniae membrane. Flow cytometry analysis showed that there was a significant difference in the mean fluorescence intensities (MFIs) between M. hyopneumoniae strain 168 treated with anti-rGAPDH serum and strain 168 treated with preimmune serum (Fig. 2D). The MFI of strain 168 treated with anti-rGAPDH serum was approximately 18.29-fold higher than that of strain 168 treated with preimmune serum. Colony blot analysis further confirmed the surface localization of GAPDH: the anti-rGAPDH serum reacted strongly with the M. hyopneumoniae strain colonies, whereas no signals were obtained after incubation with the negative serum (Fig. 2E). The results using three different methods allowed us to conclude that GAPDH is partially displayed on the surface of M. hyopneumoniae.

### GAPDH contributes to the adhesion of M. hyopneumoniae to PK15 cells.

**FIG 3:**
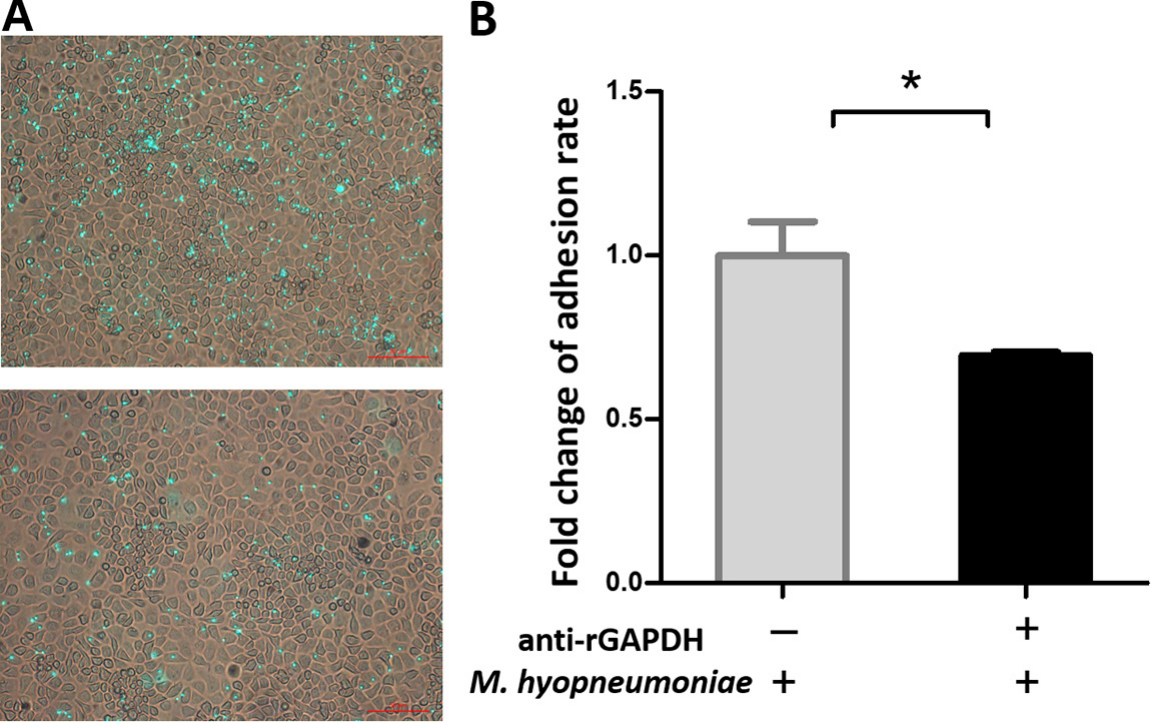
Role of rGAPDH in the adhesion of M. hyopneumoniae to PK15 cells. (A) rGAPDH adhering to PK15 cells detected using a fluorescent microsphere assay. rGAPDH (top) or BSA (bottom) was incubated with PK15 cells. Green indicates microspheres. Gray indicates PK15 cells. (B) Adhesion inhibition of rGAPDH assessed using qPCR. The adhesion rate was determined as the number of bacteria recovered in the cells incubated with anti-rGAPDH sera/number of bacteria recovered in the group incubated with preimmune sera × 100%. Data are expressed as the means ± SD from at least three experiments with samples analyzed in triplicate. *, *P* < 0.05.

The cell membrane of mycoplasma is the first component to interact with the host. Thus, surface-located proteins are very important for M. hyopneumoniae infection. To explore the potential mechanism(s) by which GAPDH affects virulence, fluorescent microspheres were used to determine whether or not rGAPDH could adhere to PK15 cells. Significantly more fluorescent microspheres were detected on the cell surface of PK15 cells incubated with rGAPDH than in cells incubated with the negative control (bovine serum albumin [BSA]) (Fig. 3A, bottom). These results provided evidence that rGAPDH could specifically bind to PK15 cells.

To further assess the role of GAPDH in M. hyopneumoniae adhesion to PK15 cells, an antibody inhibition assay was conducted. The anti-rGAPDH sera and preimmune sera were incubated with M. hyopneumoniae cells before being incubated with PK15 cells. The level of adherence of M. hyopneumoniae incubated with anti-rGAPDH sera was expressed as a percentage of the adherence of M. hyopneumoniae with preimmune sera. The results revealed that after incubation with anti-rGAPDH antibody, the adherence of M. hyopneumoniae to PK15 cells showed a 31% reduction (*P* ≤ 0.05) compared with that of M. hyopneumoniae incubated with preimmune sera (Fig. 3B). The results further indicated that GAPDH plays an indispensable role in the adherence of M. hyopneumoniae to host cells.

### rGAPDH could bind and activate plasminogen.

**FIG 4:**
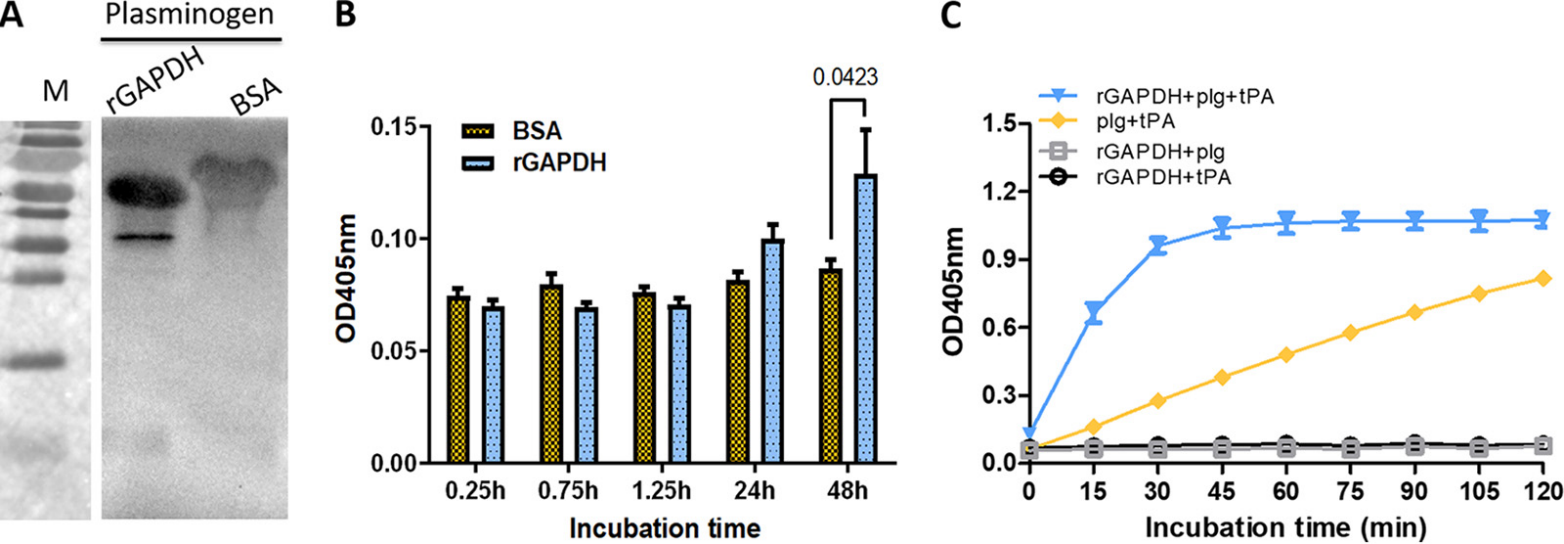
rGAPDH could bind to and activate plasminogen. (A) Far-Western blot analysis of the binding of rGAPDH to plasminogen. Bound plasminogen was detected using anti-Plg antibodies. BSA was chosen as the negative control. (B) MPAA characterization of the activity of plasminogen coated with rGAPDH or BSA. (C) Influence of rGAPDH-mediated activation of plasminogen. The activity of plasmin was evaluated by adding tPA and a chromogenic substrate and determining the OD_405_. Data are expressed as the means ± SD from at least three experiments with samples analyzed in triplicate.

The plasminogen-plasmin system plays an important role in bacterial invasion. To evaluate the interaction of GAPDH of M. hyopneumoniae with plasminogen, far-Western blotting was performed. The results showed that rGAPDH could bind to plasminogen. We observed a significantly stronger and clearer band for the interaction of rGAPDH with plasminogen than with the negative control (BSA). The analysis indicated that rGAPDH could bind to plasminogen (Fig. 4A). Several studies have demonstrated that M. hyopneumoniae infection facilitates the activation of the transformation of plasminogen to plasmin (4, 5, 7, 18). To further clarify the contribution of GAPDH to plasminogen activation, a microtiter plate adherence assay (MPAA) was carried out. The results indicated significant activation in the wells coated with rGAPDH compared with that in the wells coated with BSA upon incubation with plasminogen for 48 h (Fig. 4B). Second, we further determined the dynamic features of plasminogen activation with rGAPDH and the plasminogen activator tPA. The results showed that tPA could improve the activity of plasminogen activated by rGAPDH. The effect was time dependent and reached saturation in 1 h (Fig. 4C).

### rGAPDH binding to plasminogen could induce the degradation of the extracellular matrix.

**FIG 5:**
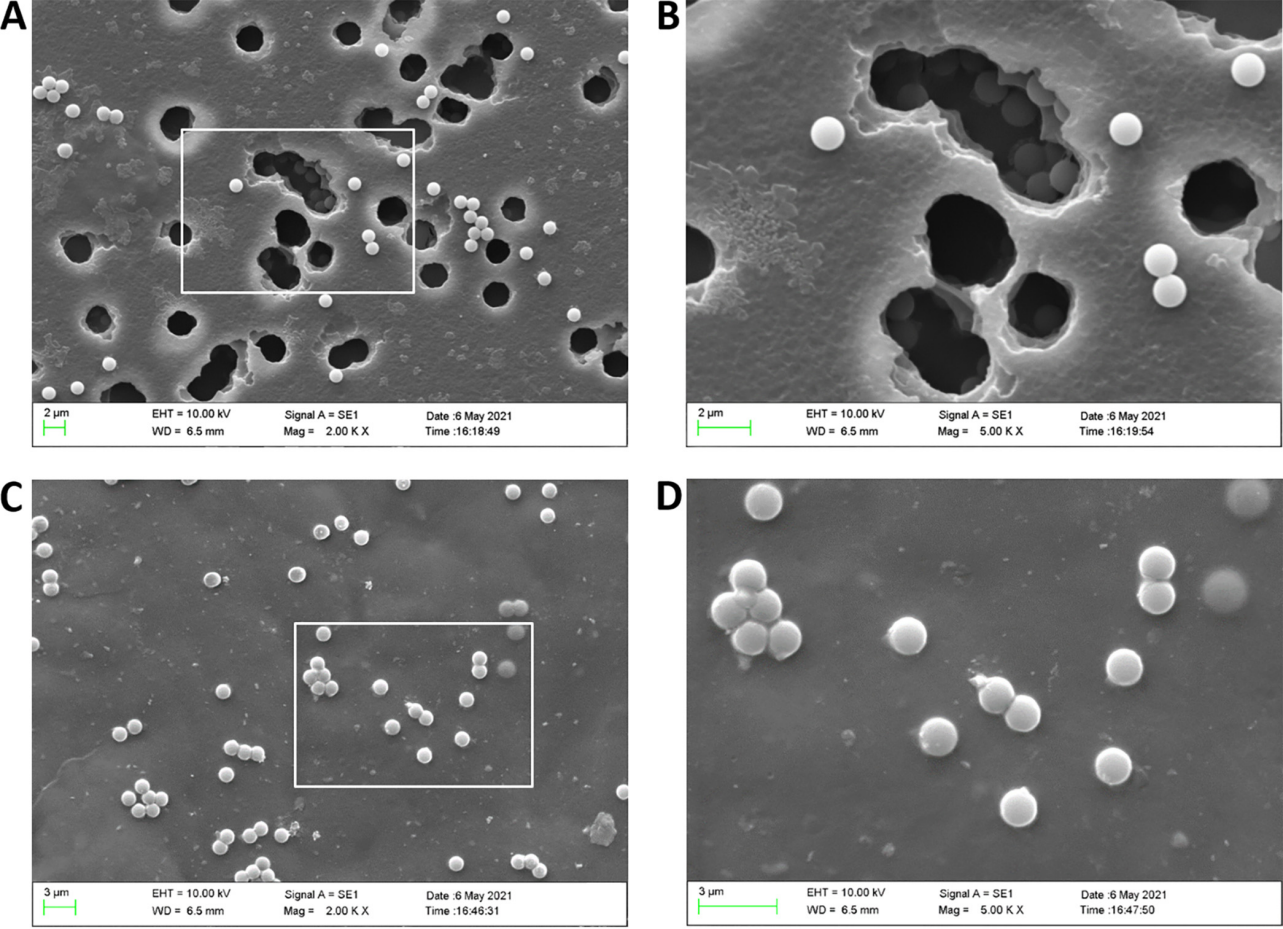
rGAPDH binding to plasminogen promotes ECM degradation. Scanning electron microscopy was used to visualize the degradation of a commercial ECM coated with rGAPDH (A and B) or BSA (C and D). Panel B is an enlarged view of a part of panel A. Panel D is an enlarged view of a part of panel C. EHT, extra high tension; WD, working distance.

The ECM is one of the most important physical barriers that restrict the spread of M. hyopneumoniae. To further clarify the function of rGAPDH binding and activation of plasminogen, the proteolytic activity of rGAPDH-activated plasminogen on Matrigel (a commercial ECM) was further assessed. The basement membrane was reconstituted by Matrigel on 3-μm filters in Transwell chambers, and the degradation of the reconstituted basement membrane was analyzed using scanning electron microscopy (SEM). Figure 5 shows significant damage to the reconstituted basement membrane, including the formation of many holes, after treatment with rGAPDH-coated and plasminogen-incubated beads. The holes in the filter membrane were exposed in some areas where the beads fell through (Fig. 5A). No degradation was observed for the BSA-coated beads (Fig. 5B). These results provide evidence that rGAPDH binds and activates plasminogen to contribute to ECM degradation.

### C-terminal lysine residues are critical for rGAPDH binding to plasminogen.

**FIG 6:**
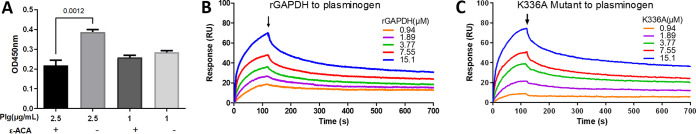
Role of C-terminal lysine residues in GAPDH binding to plasminogen. (A) ε-ACA decreased the binding of plasminogen to rGAPDH. The activity of plasmin was evaluated by adding a chromogenic substrate and determining the OD_405_. Data are expressed as the means ± SD from at least three experiments with samples analyzed in triplicate. (B and C) SPR analysis of the interaction of rGAPDH (B) and its mutant (C) with plasminogen. rGAPDH and the K336A mutant were separately injected over immobilized plasminogen. Increasing concentrations of GAPDH from 0.94 to 15.1 μM were added at a flow rate of 30 μL/min for 180 s over immobilized plasminogen. The concentrations of the proteins are consistent with the colored lines. RU, resonance units.

C-terminal lysine residues in plasminogen receptor (PlgR) molecules are usually critical for their interaction with plasminogen. To illustrate the function of C-terminal lysine residues in the interaction of rGAPDH with plasminogen, wells were coated with rGAPDH proteins and treated with different concentrations of plasminogen (1 and 2.5 μg/mL) and ε-aminocaproic acid (ε-ACA) (a lysine analog as a plasminogen activator inhibitor) or not. The results showed that the optical density (OD) value at 405 nm decreased significantly in the wells incubated with ε-ACA and higher plasminogen concentrations (Fig. 6A). To further clarify the role of C-terminal lysine residues in the interaction of GAPDH with plasminogen, a C-terminal lysine was replaced with alanine to generate a mutant GAPDH molecule (K336A). Surface plasmon resonance (SPR) analysis was used to observe real-time interactions between rGAPDH and plasminogen and between the K336A mutant and plasminogen. GAPDH could bind plasminogen in a dose-dependent and physiologically relevant manner, with an equilibrium dissociation constant (*K_D_*) of 3.764 × 10^−7^ M (Fig. 6B), while the K336A mutant showed a significantly reduced affinity, with a *K_D_* of 6.004 × 10^−7^ M (Fig. 6C). Taken together, these data support the critical role of C-terminal lysine residues in GAPDH binding to plasminogen.

## DISCUSSION

M. hyopneumoniae is a specific pathogen of pigs and is distributed worldwide. It persists in the host for a long time and is difficult to eliminate. Distinct M. hyopneumoniae strains show different degrees of virulence in pigs (22). Currently, many virulence factors have been identified for M. hyopneumoniae, and their roles include adhesion, invasion, and intracellular proliferation; however, the molecular mechanisms underlying infection and pathogenesis remain unclear. Recently, in addition to typical adhesins such as P97, P102, and P146 (5, 23–25), several metabolic enzymes, including EF-Tu, FBA, NOX, and NFOR, were described as virulence factors (8–12). They play important roles in the interaction between the host and the pathogen during *Mycoplasma* infections. These findings suggested that certain metabolic enzymes act as virulence factors, in addition to their classical roles.

An “omics” study showed that there are significant differences in the expression levels of the GAPDH gene between pathogenic strain 232 and avirulent strain J of M. hyopneumoniae. It was suggested that the higher expression level of the GAPDH gene in avirulent strain J might have developed to better cope with the rich environment (26). Meanwhile, the virulence of GAPDH of M. hyopneumoniae was predicted by website (Prediction of bacterial virulent proteins), and it shows a result of “nonvirulent”. However, the mRNA and protein expression levels of GAPDH in virulent strain 168 were significantly upregulated compared with those in its attenuated strain 168L. This result preliminarily suggested that GAPDH was correlated with the virulence of M. hyopneumoniae. Further flow cytometry analysis and colony blot analysis showed that GAPDH was partially located on the surface of M. hyopneumoniae, indicating the possibility of GAPDH’s association with the virulence of M. hyopneumoniae because surface exposure is a prerequisite for direct interactions with host cells or components.

Adhesion is a critical step in the colonization and infection of host cells by mycoplasmas and many other microorganisms. The ability for host adhesion is an important factor that reflects bacterial virulence (27). For M. hyopneumoniae, adhesion to swine tracheal epithelial cells is the first and most important step in infection. This adhesion process is complex and dynamic, with at least 35 M. hyopneumoniae proteins being previously associated with cell adhesion, including the P97/P102 paralog families and other surface proteins such as EF-Tu, GAPDH, and l-lactate dehydrogenase (LDH) that moonlight as adhesins (22, 28–30). Here, we further confirmed that GAPDH was indispensable for M. hyopneumoniae to adhere to PK15 cells. Plasminogen has been studied more extensively. The plasminogen-plasmin system in the host plays a significant role in several physiological processes such as the activation of the transformation of plasminogen to plasmin, which contributes to the degradation of the ECM (4, 19, 25), leading to tissue and structural damage and resulting in bacterial invasion of the host body. Several mycoplasmal proteins that bind to plasminogen have been found to mediate the invasion of mycoplasmas into host cells. Therefore, plasminogen-binding proteins play a critical role in the pathogenic process of mycoplasmas (13, 31–34). GAPDH is a widely studied plasminogen receptor in bacteria. It has been reported in many bacteria such as Streptococcus pneumoniae (35), E. coli (36), Clostridium perfringens (37), and *M. hyorhinis* (34). Among mycoplasma species, GAPDH of M. pneumoniae is the most widely studied. Previous reports have confirmed that M. pneumoniae GAPDH can bind to plasminogen (14, 38, 39). In this study, we found that rGAPDH could specifically bind to plasminogen, which indicated that the association of GAPDH with the virulence of mycoplasmas might be mediated via binding to plasminogen.

The basement membrane under porcine respiratory epithelial cells is one of the most important physical barriers. Thus, the degradation of the ECM or basement membrane is critical in the process of invasion by bacteria (13, 22). rGAPDH of *M. hyorhinis* has been reported to be able to bind plasminogen, and the bound plasminogen could be activated by tPA to form the active serine protease plasmin, which degrades the ECM (34). However, whether GAPDH of M. hyopneumoniae has similar functions and is responsible for the virulence of M. hyopneumoniae was unknown. In the present study, we demonstrate that rGAPDH of M. hyopneumoniae could bind to plasminogen. Next, plasminogen was activated by tPA to form the active serine protease plasmin. This resulted in the degradation of the ECM. The binding activity of rGAPDH was significantly inhibited by the addition of ε-ACA, and the importance of the C-terminal lysine of rGAPDH in the affinity for plasminogen was proven using a lysine residue mutant. Nevertheless, the function of lysine in the interaction with plasminogen requires further confirmation.

In conclusion, we reveal that GAPDH is a multifunctional protein present at a higher abundance in a highly virulent M. hyopneumoniae strain, which participates in cytoadhesion and functions as a receptor for plasminogen binding by C-terminal lysines. We speculate that GAPDH might act as a virulence factor of M. hyopneumoniae by hijacking host plasminogen. This is an important virulence mechanism that promotes adhesion and ECM degradation, which could partially explain previous observations that M. hyopneumoniae is not just a respiratory tract-inhabiting pathogen.

## MATERIALS AND METHODS

### Ethical statement.

Animal Experiments have been approved by Jiangsu Academy of Agricultural Sciences (License No. SYXK (Su) 2015-0019). All the experimental procedures were conformed to the regulations on the Administration of Experimental Animals in Jiangsu Province (Government Decree No. 45).

### Culture of bacteria and cells.

Highly virulent M. hyopneumoniae strain 168 and its attenuated highly passaged strain 168L were stored in our laboratory (Jiangsu Academy of Agricultural Sciences). M. hyopneumoniae strain 168 is a pathogenic strain that was originally isolated from an Er-Hua-Lian pig that showed typical clinical signs in 1974 (9). The stable attenuated strain 168L was obtained by serial cultivation in KM2 cell-free medium (a modified Friis medium) containing 15% (vol/vol) swine serum at 37°C in a 5% CO_2_ incubator for over 300 passages. Escherichia coli strain BL21(DE3) was cultured in Luria-Bertani (LB) broth or on solid medium containing 1.5% agarose supplemented with 0.1 μg/mL ampicillin. The pET-32a expression vector was obtained from Novagen (Merck, Germany). PK15 is a porcine epithelial cell line derived from a normal pig kidney, which was purchased from the American Type Culture Collection (ATCC) (Manassas, VA, USA). Cells were maintained at 37°C with the provision of 5% CO_2_ in Dulbecco’s modified Eagle medium (DMEM) supplemented with 10% fetal bovine serum (FBS).

### Reverse transcription-quantitative PCR.

Reverse transcription-quantitative PCR (RT-qPCR) was performed to analyze the expression of the gene at the transcriptional level. Highly virulent M. hyopneumoniae strain 168 and its attenuated strain 168L were cultured under the above-mentioned culture conditions at 37°C for 24, 36, and 48 h and then harvested by centrifugation at 15,000 × *g* for 20 min at 4°C. Total RNA was extracted using a total RNA extraction kit (catalog no. R1200-100; Solarbio, Beijing, China). The purity and concentration of the total RNA were measured using the ratio of the absorbance at 260/280 nm. Next, 1 μg of RNA was reverse transcribed into cDNA in a 20-μL reaction mixture volume using HiScript II Q Select RT SuperMix for qPCR (+gDNA wiper) (catalog no. R223-01; Vazyme, Nanjing, China) according to the manufacturer’s protocol. The relative mRNA expression levels of the GAPDH gene were determined using the cDNA as the template in the qPCR step of the RT-qPCR protocol using the ABI 7500 real-time PCR system (Applied Biosystems, Foster City, CA, USA) with the HiScript II one-step RT-qPCR SYBR green kit (catalog no. Q221-01; Vazyme). The total volume of the reaction mixture was 20 μL. The *gatB* gene of M. hyopneumoniae was selected as the internal control, and the thermocycler program was the same as the one described previously (40). The relative mRNA levels were determined using the 2^−ΔΔ^*^CT^* method (41). The PCR primers used in this study are listed in Table 1.

**TABLE 1:** Primers for RT-qPCR and qPCR

Analysis and gene target or primer/probe name	Primer direction, sequence (5′–3′)^*a*^
RT-qPCR of GAPDH gene expression at the transcriptional level	
GAPDH	F, TTGGCACTTCGTCGTCTTT
	R, CCATGGGCGGAATCATACTT
*gatB*	F, AATGGATCACTTCGTGCTGATA
	R, TCAAGTTCGGCGGCTTT
qPCR of M. hyopneumoniae	
MHP 183	F, CCAGAACCAAATTCCTTCGCTG
	R, ACTGGCTGAACTTCATCTGGGCTA
MHP-P	FAM-AGCAGATCTTAGTCAAAGTGCCCGTG-BHQ

a F, forward; R, reverse; FAM, 6-carboxyfluorescein; BHQ, black hole quencher.

### Expression and purification of rGAPDH.

The entire GAPDH gene was generated synthetically based on the sequence of M. hyopneumoniae strain168 (GenBank accession no. CP002274.1). Meanwhile, the point mutant of GAPDH formed by replacing the C-terminal lysine at position 366 with alanine (K366A) was generated using a Mut Express MultiS fast mutagenesis kit (Vazyme). All genes were inserted into the pET-32a vector between the XhoI and BamHI cleavage sites and were constructed by Genscript Biotech (Nanjing, China). The recombinant plasmid pET32a-GAPDH was transformed into E. coli BL21(DE3), and the plasmid from a selected single clone was verified by sequencing by Sangon Biotech (Shanghai, China). E. coli cells at log phase (optical density at 600 nm [OD_600_] of 0.6 to 0.8) were treated with isopropyl-β-d-thiogalactopyranoside (IPTG) at a final concentration of 0.5 mM for 22 h at 16°C to induce expression. The recombinant protein was purified using Ni resin FF (Genscript, Nanjing, China), and the buffer was exchanged and concentrated using a centrifugal filter (Millipore, Billerica, MA, USA). The purified rGAPDH protein was electrophoresed using 12% sodium dodecyl sulfate-polyacrylamide gel electrophoresis (SDS-PAGE) to assess its purity. The protein concentration was measured using a bicinchoninic acid (BCA) protein assay kit (Beyotime, Jiangsu, China). Finally, the obtained rGAPDH was stored at −80°C until use.

### Preparation of polyclonal antibodies against rGAPDH.

Polyclonal antibodies were raised against rGAPDH by subcutaneously immunizing New Zealand White rabbits (about 2 to 2.5 kg). The purified rGAPDH protein (1 mg/rabbit) emulsified with Freund’s complete adjuvant (1:1, vol/vol; Sigma, USA) was injected into the back of the rabbit subcutaneously; each rabbit was immunized three times at 14-day intervals. Antisera were collected 1 week after the final injection. Western blotting was performed to determine the reactivity and specificity of the prepared polyclonal antibodies. Finally, all anti-rGAPDH sera were dispensed into 1.5-mL eppendorf tubes and stored at −80°C until use.

### Western blot analysis.

The cellular localization of GAPDH in M. hyopneumoniae was investigated according to a protocol described previously by Zhao et al. (27). Briefly, the membrane proteins of M. hyopneumoniae were extracted by using a proteoprep membrane extraction kit (Sigma, Roedermark, Germany), according to the manufacturer’s instructions. Meanwhile, cultures of M. hyopneumoniae 168 and 168L were harvested by centrifugation at 11,000 rpm for 20 min at 4°C, washed, and resuspended in phosphate-buffered saline (PBS) to prepare whole-cell protein. The BCA protein assay kit was used to measure the concentration of the extracted protein.

Western blotting was used to determine the specificity of anti-rGAPDH sera, the protein expression level of GAPDH in highly virulent M. hyopneumoniae strain 168 and its attenuated strain 168L, and the location of GAPDH in M. hyopneumoniae. For Western blot analysis, all protein samples were adjusted to 1 mg/mL after the BCA protein assay. Samples (10 μg) were separated using 12% SDS-PAGE and then transferred onto polyvinylidene difluoride (PVDF) membranes (Millipore, Darmstadt, Germany). After blocking with 5% skimmed milk in Tris-buffered saline–Tween 20 (TBST) buffer (20 mM Tris-HCl [pH 8.0], 150 mM NaCl, and 0.1% Tween 20) for 2 h at 37°C, the membrane was stained with Ponceau S stain as the loading control. After washing three times, the membrane was incubated with anti-rGAPDH sera (diluted 1:1,000) for 1.5 h at 37°C and washed with TBST buffer three times for 15 min each. The membrane was then incubated with horseradish peroxidase (HRP)-conjugated goat anti-rabbit IgG (1:5,000; Boster, Wuhan, China) for 1.5 h at 37°C. Thereafter, the membrane was washed three times for 15 min each. The immunoreactive protein bands were visualized using the Chemister High-sig ECL Western blotting substrate (Tanon, Shanghai, China) according to the manufacturer’s instructions.

### Flow cytometry analysis.

Flow cytometry was used to determine whether GAPDH was displayed on the surface of M. hyopneumoniae. Highly virulent M. hyopneumoniae strain 168 was cultured in KM2 medium containing 15% (vol/vol) swine serum at 37°C until the KM2 medium turned orange-yellow (10^8^ colony-changing units [CCU]). The pellets were harvested by centrifugation, washed twice with PBS, and then incubated with anti-rGAPDH serum at a 1:100 dilution (preimmunized rabbit serum was used as a negative control) for 1 h at 37°C. Fluorescein isothiocyanate (FITC)-conjugated goat anti-rabbit IgG at a 1:500 dilution was used as the secondary antibody for 1 h at 37°C. Finally, the fluorescence intensity was determined using a flow cytometer (BD Accuri C6; BD Biosciences, San Jose, CA, USA). The mean fluorescence intensity (MFI) of M. hyopneumoniae incubated with anti-rGAPDH serum was expressed as a percentage of that of M. hyopneumoniae incubated with preimmune serum.

### Colony blot analysis.

The colony blot technique was used to confirm if GAPDH was present at the surface of mycoplasma colonies. Strain 168 was cultured in KM2 medium containing 15% (vol/vol) swine serum at 37°C until the KM2 medium turned orange-yellow. The culture was harvested by centrifugation at 11,000 rpm for 20 min at 4°C and diluted with KM2 medium at 1:1,000, and 200 μL of the culture was spread evenly onto the surface of KM2 solid medium and incubated at 37°C until mycoplasma colonies could be observed. PVDF membranes were gently placed onto mycoplasma colonies on the surface of the agar plates and pressed gently to ensure that the membrane fit completely. After 5 min, the PVDF membranes were treated using the same steps as the ones described above for Western blot analysis. Preimmune serum was used instead of anti-rGAPDH serum as the negative control.

### Fluorescent microsphere analysis.

The fluorescent microsphere method was used to determine the ability of recombinant proteins to adhere to PK15 cells. A commercial polystyrene latex bead stock solution (30%) (LB11; Sigma-Aldrich, USA) was resuspended and diluted to a 1% microsphere suspension with 25 mM 2-(*N*-morpholino)ethanesulfonic acid (MES) buffer (pH 6.1), filtered through a 40-μm cell sieve, centrifuged at 5,000 × *g* for 5 min, and washed three times with MES. Next, 200 μL rGAPDH and BSA (2 mg/mL) were incubated with the microspheres, separately, overnight at 4°C. After centrifugation at 5,000 × *g* for 5 min, the supernatant of the microsphere protein suspension was carefully discarded, and the beads were washed three times with MES. The microspheres were then blocked with MES containing 5% BSA and incubated at room temperature for 1 h. Cultured PK15 cells were blocked with PBS buffer containing 5% BSA at 37°C for 30 min. The blocked microspheres were washed with MES and resuspended in DMEM. The blocked cells were washed three times with PBS buffer and incubated with microspheres at 37°C for 5 h. After washing five times with PBS buffer, the cells were observed using fluorescence microscopy.

### DNA isolation and qPCR.

A qPCR test was performed to analyze the adhesion inhibition of anti-rGAPDH sera. PK15 cells were seeded onto 24-well plates and cultured to 80% confluence. Freshly cultured M. hyopneumoniae strain 168 cells (10^8^ CCU) were harvested by centrifugation at 15,000 × *g* for 20 min at 4°C, washed twice with PBS, and then incubated with anti-rGAPDH sera or preimmune sera (1:20 dilution) in 1 mL PBS at 37°C for 30 min. Three independent replicates were performed for each sample. M. hyopneumoniae pellets were harvested and resuspended in DMEM with 2% FBS at 10^8^ CCU/mL. Confluent PK15 cells were washed twice with PBS and incubated with 500 μL of the M. hyopneumoniae suspension at 37°C for 6 h. The dissociated M. hyopneumoniae bacteria were removed by washing them five times with PBS. The cells in the wells were digested using 0.25% trypsin and collected. Genomic DNA was extracted using a Tiangen (Beijing, China) bacterial DNA kit, and qPCR was performed using a quantstudio real-time PCR system according to a previously reported method, with slight modifications (42). The primers used in this study are listed in Table 1. The data were analyzed using Student’s *t* test in SPSS 20.0 (IBM Corp., Armonk, NY, USA). For all tests, a *P* value of ≤0.05 was considered statistically significant. The assays were performed in triplicate.

### Far-Western blot analysis.

Far-Western blotting was performed to explore whether GAPDH could bind to plasminogen. rGAPDH (20 μg) or BSA (negative control) was separated using 12% SDS-PAGE and transferred to PVDF membranes (9). After blocking with 5% (wt/vol) skimmed milk for 2 h at 37°C, the membrane was incubated with 10 μg/mL plasminogen (Boster) at 37°C for 2 h, followed by incubation with 5 μg/mL anti-plasminogen antibody (Boster). HRP-conjugated goat anti-rabbit IgG (Boster) (diluted 1:5,000) was used as the secondary antibody. Finally, the membrane was visualized using the Chemister High-sig ECL Western blotting substrate (Tanon) according to the manufacturer’s instructions.

### Microtiter plate adherence assay (MPAA).

Ninety-six-well microtiter plates were coated with rGAPDH (30 μg/mL; 100 μL/well) overnight at 4°C. Following blocking with 5% BSA, the plates were incubated with 100 μL of plasminogen (10 μg/mL) at 37°C for 3 h. Following washing, 100 μL of tissue-specific plasminogen activator (tPA; Sigma-Aldrich, St. Louis, MO, USA) was diluted to 500 ng/mL with PBS buffer and incubated at 37°C for 2 h. Following washing, d-Val-Leu-Lys *p*-nitroaniline dihydrochloride (Sigma-Aldrich), a plasminogen-specific substrate, was added at a final concentration of 0.4 mM, and the mixture was incubated at 37°C. After 0.25, 0.75, 1.25, 24, and 48 h, activity was estimated from the OD values measured at 405 nm. Wells without tPA were set up to determine the ability of the rGAPDH protein to directly activate plasminogen.

### Enzyme-linked immunosorbent assay.

The kinetics of plasminogen activation by tPA with rGAPDH (or not) was further measured using an enzyme-linked immunosorbent assay (ELISA). rGAPDH (20 μg/mL) and plasminogen (20 μg/mL) were mixed and incubated at 37°C for 1 h. The mixture was added to a flat-bottom 96-well plate. Next, 500 ng/mL tPA was added to the wells, the mixture was incubated for 15 min, 0.4 mM substrate was added, and the plates were incubated at 37°C. The OD_405_ was measured every 15 min from 15 min to 120 min. Wells containing only plasminogen with tPA, rGAPDH with plasminogen, or rGAPDH with tPA served as the controls. The experiments were performed in triplicate.

Meanwhile, an ELISA was also applied to determine the role of C-terminal lysine residues in the binding of GAPDH to plasminogen. The methods were as same as the ones described above except that the plates were incubated with 100 μL of plasminogen (2.5 μg/mL or 12.5 μg/mL) for 3 h at 37°C in the presence or absence of 200 mM ε-aminocaproic acid (ε-ACA) (Sigma-Aldrich) (a lysine analog). After 48 h, the activity was estimated from the OD values measured at 405 nm.

### Scanning electron microscopy.

Scanning electron microscopy (SEM) was used to examine the ability of rGAPDH-bound plasminogen to degrade the ECM (Matrigel; Corning Inc., Corning, NY, USA). Matrigel was first diluted 1:3 in ice-cold PBS, spread over a 3-μm transparent membrane insert (Corning Inc.), gelatinized at 4°C for 30 min, and then dried overnight at 37°C. Before the degradation assay was performed, gels were rehydrated with 70 μL of PBS for 1 h at 37°C. Polystyrene beads were used to immobilize the proteins. According to the manufacturer’s instructions, rGAPDH or BSA was passively adsorbed onto the particle surface. Briefly, 1% (vol/vol) beads were suspended in 1 mL of the rGAPDH or BSA solution (1.5 mg/mL) and incubated overnight at 4°C. After washing, the beads were blocked with 5% BSA. Following PBS buffer washing, the beads were incubated with 10 μg/mL plasminogen for 3 h at 37°C. Thereafter, the beads were washed with sterile PBS to remove unbound plasminogen. Following incubation with tPA (500 ng/mL) for an additional 2 h at 37°C, the beads were washed and resuspended in 1 mL of PBS. The resuspended beads were added to the upper compartment of a Transwell chamber, while the lower compartment contained 700 μL of PBS. The chamber was incubated at 37°C for 40 h. The obtained Transwell membranes were fixed with 2.5% glutaraldehyde and examined by SEM using a Zeiss EVO-LS10 SEM instrument (Carl Zeiss, Oberkochen, Germany).

### Surface plasmon resonance.

Further investigation of the kinetics of the interaction between rGAPDH and plasminogen was performed in real time by surface plasmon resonance (SPR) experiments using a Biacore X100 Plus instrument (GE Healthcare, Chicago, IL, USA). Plasminogen was diluted to 10 μg/mL in 10 mM sodium acetate (pH 4.0) and covalently linked to the carboxymethyl glucan matrix of the CM5 sensor chip as a ligand using an amine coupling kit (Biacore AB, Uppsala, Sweden). The resonance unit (RU) value generated by the immobilized soluble protein was 600. Binding kinetics were determined by increasing the concentrations (0 to 15 μM) of the analyte (rGAPDH and the K336A mutant) in running buffer (10 mM HEPES, 150 mM NaCl, 3 mM EDTA [0.05%, vol/vol]), Binding kinetics analysis was measured using increasing concentrations (0–15 μM) of the analyte (rGAPDH and the K336A mutant) in running buffer (HBS-EP) consisting of 10 mM HEPES, 150 mM NaCl, 3 mM EDTA, and 0.05% (v/v) surfactant P20 (Biacore AB) at a flow rate of 30 μL/min for 180 s over immobilized plasminogen at 20°C. The dissociation phase was monitored for 1,000 s by allowing the buffer to flow through the chip. Binding kinetics were analyzed manually using Biacore X100 control software (9, 15).
